# Influence of socioeconomic status on changes in body size and physical activity in ageing black South African women

**DOI:** 10.1186/s11556-018-0196-8

**Published:** 2018-04-26

**Authors:** Philippe Jean-Luc Gradidge, Shane A. Norris, Richard Munthali, Nigel J. Crowther

**Affiliations:** 1 Centre for Exercise Science and Sports Medicine, Faculty of Health Sciences, University of the Witwatersrand, Johannesburg, South Africa; 2 MRC/Wits Developmental Pathways for Health Research Unit, Faculty of Health Sciences, University of the Witwatersrand, Johannesburg, South Africa; 3 Department of Chemical Pathology, National Health Laboratory Service, Faculty of Health Sciences, University of the Witwatersrand, Johannesburg, South Africa

**Keywords:** Body mass index, Waist circumference, Socioeconomic status, Physical activity, Sitting time, Urban, African women

## Abstract

**Background:**

The increasing prevalence of obesity in sub-Saharan African women is not well understood, and black South African women in the region are particularly vulnerable. This study aimed to examine whether the relationship of socioeconomic status (SES) with changes in body mass index (BMI) and waist circumference (WC) is mediated by physical activity in ageing African women.

**Methods:**

In a longitudinal analysis of the 518 caregivers associated with the Birth to Twenty Plus study, the role of SES associated with 10-year changes in BMI and WC was tested using structural equation modelling (SEM). The degree of mediation of moderate-vigorous physical activity (MVPA) and sitting time in this association was also assessed.

**Results:**

The prevalence of obesity increased significantly from baseline to follow-up (*p* < 0.0001). In the SEM models, baseline SES had a direct positive effect on changes in BMI (β, 95% CI, 0.02 (0.005 to 0.04), and a direct negative effect on changes in MVPA (β, 95% CI, − 3.81 (− 6.92 to − 0.70). Baseline MVPA had a direct negative effect (β, 95% CI, − 0.002 (− 0.003 to − 0.0003) and indirect positive effect via change in MVPA (β, 95% CI, 0.01 (0.0001 to 0.001) on change in WC.

**Conclusions:**

Our study demonstrates the role and interaction of sociodemographic and behavioural predictors of obesity, and suggests a multifaceted approach to management of the crisis in communities of ageing urban African women.

**Electronic supplementary material:**

The online version of this article (10.1186/s11556-018-0196-8) contains supplementary material, which is available to authorized users.

## Background

Obesity is multifaceted and complex, driven largely by factors associated with obesogenic urban environments [1]. Within these settings, the poor seem to have a greater burden of obesitycompared with the more affluent [2]. Recent evidence demonstrates that the prevalence of obesity has doubled since 1980 and currently affects more than 604 million adults globally [3]. These data indicate a trend for obesity to continue increasing, especially in women living in low- and middle-income countries. The estimates of obesity are higher in north and southern Africa than the global prevalence of obesity [4], with a recent study demonstrating that the increase in severe obesity from 1975 to 2014 in South African women was higher than those of more affluent countries such as France and the United Kingdom [5]. These trends in obesity are worrying for African populations, particularly as obesity increases the risk of associated non-communicable chronic diseases and certain types of cancers [6], in addition to lowering life expectancy by increasing the risk of early mortality [7]. Being obese is also associated with lower health-related quality of life and functionality, and is especially important in the context of an increasing ageing population with excess fat accumulation [8].

In the sub-Saharan African region, it is black South African women who are the most vulnerable to overweight and obesity [9]. The underlying causes of the excess fat accumulation, particularly in the visceral region of ageing women are uncertain, however an increased risk of cardiometabolic diseases has been observed [10], in addition to an associated lower physical activity energy expenditure in older subjects compared with younger subjects [11] . In the South African setting where disparities are still evident, socioeconomic status (SES) may also have a role in the weight gain of African women. Data show that black South African women are generally poorer than other ethnic groups in the country and have limited access to adequate healthcare [12]. Moreover, previous cross-sectional studies conducted in South Africa [13–15] and other countries in the sub-Saharan African region [16, 17] have observed a positive association between higher body mass index (BMI) and improved SES. In addition, the contribution of SES and behaviours such as increased sitting time and physical inactivity on adiposity in high-income countries are well documented [18, 19], however evidence is limited on how these factors mediate and influence anthropometric changes in African populations. Therefore, the aims of this study were two-fold: (1) to determine whether SES correlates with anthropometry (both at baseline and at 10 year follow-up); and (2) to determine whether the interaction of SES with anthropometry is mediated by either moderate-vigorous physical activity (MVPA) or sitting time by using structural equation modelling (SEM).

## Methods

### Participants and setting

This longitudinal study included black South African urban-dwelling women from Soweto, Johannesburg [20]. These women were caregivers of participants from the Birth to Twenty Plus (Bt20) cohort, a study which started in 1990 with a sample of 3273 subjects [21, 22]. Women were enrolled in their second and third trimester of pregnancy through public health facilities and interviewed regarding their health and social history and current circumstances. Attrition over two decades has been comparatively low, mostly occurring during the children’s infancy and early childhood [23]. The Bt20 sample is representative of families that have remained residents of Soweto for over 20 years, with equal numbers of male and female participants. Bt20 also monitors the health of the biological mothers or caregivers of the children and it is from these subjects that the study cohort was drawn. The baseline measurements for the current study were collected in 2002/3 on 1249 adult women (aged ≥ 18 years), and the follow-up data collection wave was completed 10 years later (2012/13) on 702 women. This resulted in 518 women having body composition data at both time points, due to drop-out. Ethical approval was granted by the University of the Witwatersrand (Ethics certificate number: M110627) and all participants provided written consent.

### Biological variables

Body weight (kg) was measured to the nearest 0.1 kg using a digital weighing scale (Dismedinc., Anjou, Canada) and standing height was measured to the nearest mm using a wall stadiometer (Holtain Ltd., Crosswell, UK). The participants wore minimal clothing and did not have shoes on during the measurements. Trained research assistants conducted the measurements, and the coefficients of variation for body weight and standing height measurements were both < 1%. Body mass index (BMI, kg.m^− 2^) was calculated, and obesity was defined as BMI ≥ 30 kg.m^− 2^ [24]. Using a flexible, but inelastic measuring tape, a measurement of the waist circumference was taken at the narrowest part of the trunk, horizontally, while the participants were standing with arms at the side, relaxed abdomen, and feet together [24]. The coefficients of variation for waist and hip circumference measurements were < 2%. Central obesity was defined as waist circumference ≥ 80 cm.

### Moderate-vigorous physical activity and sitting time

Physical activity and sitting time was assessed using the interviewer-administered Global Physical Activity Questionnaire or GPAQ, developed for global physical activity surveillance [25]. Total moderate-vigorous physical activity (MVPA) minutes per week were calculated from the accumulative occupation-, travel-, and leisure-related time physical activity. Sitting time (mins/wk) was used as a proxy for sedentary behaviour.

### Socioeconomic status and related variables

A questionnaire was used to determine SES by using twelve household commodities to generate an SES index. The tool has been validated for use in South Africa by Griffiths et al. [26]. The use of asset indices have been acknowledged to the valuable proxy measures of SES in epidemiological studies [27]. The household indices used in this study included electricity, television, radio, motor vehicle, refrigerator, washing machine, telephone, video machine, microwave, analog television channel decoder (MNET), satellite television (DSTV), and mobile phone. These twelve household commodities were ranked in order of value and an overall SES score was then calculated using the ranks. The overall SES score ranged from 0 (ownership of no assets) to 78 (ownership of all 12 assets). The sample was split into 2 groups by SES median score, i.e. 30, and differences between these 2 groups were determined for all measured variables. Living arrangement was categorised and coded as: code 1 for ‘living together’ (married or co-habiting), or code 2 for ‘single’ (divorced, separated, widowed, and not married). Highest level of education was collected at baseline and 10-year follow-up and coded as ‘1’ for completion of primary school, ‘2’ for incomplete high school, and ‘3’ for completion of high school.

### Statistical analysis

Continuous parametric data are presented as mean ± SD or are displayed as median (interquartile range [IQR]) if their distribution was non-parametric. The physical activity measures, MVPA and sitting time were log transformed to normality. Data was compared between baseline and follow-up time points using Student’s paired t test. Categorical, baseline data are presented as mean (95% CIs). Student’s non-paired t test was used to compare baseline values for age, anthropometric and physical activity measures between groups with SES scores above or below the median value of 30.

Structural equation modelling (SEM) was used to test and estimate the role of baseline SES (exogenous factor) on change in BMI, change in waist circumference, change in sitting time, and change in MVPA (endogenous factors). In the SEM model for change in BMI and change in MVPA, the potential mediators included baseline MVPA, change in MVPA (for change in BMI model only). Likewise, in the model for change in waist circumference and change in MVPA, baseline sitting time and change in sitting time (for the change in waist circumference model only) were the potential mediators. In the model for change in BMI and change in sitting time, baseline sitting and change in sitting time (only in the change in BMI model) were included as potential mediators. In the model for change in waist circumference and change in MVPA, baseline MVPA and change in MVPA (only in the change in waist circumference model) were included as potential mediators. Age was treated as a confounding variable in all the SEM models. Univariate analysis, using Spearman Rank Order correlations, were conducted to determine the association of SEM parameters with the dependent variables. Direct, indirect and total effects were computed and recoded, and the proportion of total effects mediated was calculated for assessing mediation roles. Different goodness of fit indices was used to evaluate the best fitting model. Chi-squared test, Root mean squared error of approximation (RMSEA), Comparative fit index (CFI), Tucker-Lewis index (TLI) and Standardized root mean squared residual (SRMR) were calculated and recorded [28]. We employed the Hu and Bentler’s Two-Index Presentation Strategy (1999) combination rule with cut off values for CFI (≥ 0.90), SRMR (≤ 0.09), RMSEA (≤ 0.06) included for best fit [28].

## Results

### Study population

Table 1 displays the study population’s baseline and 10-year follow-up characteristics. Mean age at baseline was 41.1 ± 5.8 years and at follow-up age was 49.3 ± 5.3 years. BMI increased significantly from 31 ± 6.7 kg.m^− 2^ at baseline to 33.3 ± 7.4 kg.m^− 2^ at follow-up (*p* < 0.0001), while waist circumference similarly increased from 88 ± 13.3 at baseline to 98.9 ± 14.6 at follow-up (*p* < 0.0001). Obesity (BMI ≥ 30 kg.m^− 2^) and central obesity increased significantly from baseline to 10-year follow-up (*p* < 0.0001). Sitting time decreased significantly from baseline to follow-up (*p* < 0.0001). Women in the lower SES group were older (41.7 ± 7.83 vs 40.7 ± 8.01, respectively, *p* = 0.04) and had a lower change in BMI compared with women above the median for SES (1.80 ± 3.59 vs 2.94 ± 3.90, respectively, *p* = 0.001). No other differences between the two SES groups were observed. Sensitivity tests of the baseline study characteristics were conducted to check for selection and attrition bias. As shown in Additional file 1, there were no significant differences between those at baseline and at 10-year follow-up using the baseline study characteristics. Just under half of the sample were living together, 46.7 (43.7; 49.4), while the mean SES score was 32.7 ± 16.8.

**Table 1:** Sample characteristics of urbanised black South African women living in Soweto

	Baseline	Follow-up	Net change	*p*-value
Age (years)	41.4 ± 5.8	49.3 ± 5.3	+ 7.9	< 0.0001
Height (m)	1.58 ± 0.05	–	–	–
Weight (kg)	76.1 ± 15.1	80.5 ± 16.3	+ 4.4	< 0.0001
BMI (kg.m^−2^)	31 ± 6.7	33.3 ± 7.4	+ 2.3	< 0.0001
BMI ≥ 30 kg.m^− 2^, %	52.2 (48.1, 56.4)	67.4 (63.4, 71.2)	+ 15.2	< 0.0001
WC (cm)	88 ± 13.3	98.9 ± 14.6	+ 10.9	< 0.0001
Central obesity, WC ≥ 80 cm, %	72 (68, 75.6)	90 (87.2, 92.2)	+ 18	< 0.0001
MVPA (mins./week)	350 (150, 1230)	240 (60, 1035)	−110	0.57
Sitting time (mins./week)	1260 (840, 2100)	690 (510, 915)	− 570	< 0.0001

Data presented as mean ± SD or percentage (%) (95% CIs) or median (interquartile range); *+* increase; − decrease

*BMI* body mass index; *MVPA* moderate-vigorous physical activity; *WC* waist circumference; Paired t-tests were used to describe the difference in baseline and follow-up values

At baseline 12.5% (9.94, 13.6%) attended primary school, 70.3% (67.7, 72.8%) did not complete high school, and only 17.2% (15.2, 19.4%) completed high school. The 10-year, follow-up data showed that there was an increase in the amount of women who completed high school (*p* < 0.0001) (12.5% (10.7, 14.5%) completed primary school, 57.0% (52.8, 61.1%) did not complete high school, and 30.7% (27.0, 34.7%) completed high school). The women who completed high school were younger than those who only completed school at an elementary level (40.3 ± 6.79 vs 46.4 ± 7.86 years; *p* < 0.0005). There were no other differences between the education status groups for body composition or physical activity indicators at baseline or absolute change.

In a univariate analysis of the parameters used in the SEM models, there was a positive correlation between baseline SES and change in BMI (*r* = 0.13, *p* = 0.002), and a negative correlation between age and change in BMI (*r* = − 0.14, *p* = 0.001) (see Table 2). Change in waist was negatively correlated with baseline waist (*r* = − 0.19, *p* < 0.0001) and age (*r* = − 0.09, *p* = 0.03). Change in MVPA was negatively correlated with baseline MVPA (*r* = − 0.68, *p* < 0.0001), and baseline sitting time (*r* = − 0.12, *p* = 0.01), while positively correlated with change in sitting time (r = 0.13, 0.006). Changes in sitting time was positively correlated with baseline sitting time (*r* = 0.92, *p* < 0.0001), while negatively correlated with baseline MVPA (*r* = − 0.14, *p* = 0.005).

**Table 2:** Correlation between physical activity, socioeconomic status parameters and endogenous factors used in the structural equation modelling

Parameter	BMI^b^	WC^b^	MVPA^b^	Sitting time^b^
Age^a^	− 0.14 (0.001)	− 0.09 (0.03)	− 0.01 (0.82)	0.02 (0.67)
BMI^a^	− 0.07 (0.12)	–	− 0.02 (0.78)	0.06 (0.62)
WC^a^	–	− 0.19 (< 0.0001)	− 0.02 (0.61)	0.04 (0.46)
MVPA^a^	−0.03 (0.54)	− 0.01 (0.14)	− 0.68 (< 0.0001)	−0.14 (0.005)
MVPA^b^	−0.03 (0.54)	−0.01 (0.76)	–	0.13 (0.006)
Sitting time^a^	0.02 (0.64)	0.07 (0.16)	−0.12 (0.01)	0.92 (< 0.0001)
Sitting time^b^	−0.01 (0.76)	−0.06 (0.25)	0.13 (0.006)	–
SES score^a^	0.13 (0.002)	0.08 (0.07)	−0.08 (0.09)	0.04 (0.47)

Data are presented as r (*p*-value)

*BMI* body mass index; *MVPA* moderate-vigorous physical activity; *SES* socioeconomic status; *WC* waist circumference

^a^Baseline values

^b^Change values

### Structural equation modelling

The SEM models for changes in BMI, changes in WC, and changes in physical activity measures and the interaction with baseline SES are shown in Table 3. Baseline SES had a direct positive effect on change in BMI and a direct negative effect on change in MVPA (*p* < 0.05). Baseline SES had a direct negative effect (via baseline WC) on change in MVPA (*p* < 0.05). Baseline MVPA had an indirect negative effect (via change in MVPA) on change in BMI, and an indirect negative effect on change in WC (via change in MVPA). Baseline MVPA had a direct negative effect on change in WC (*p* < 0.05), and a direct negative effect on change in MVPA (*p* < 0.0001). Baseline sitting time had an indirect negative effect (via change in sitting time) on change in BMI and change in WC (*p* < 0.0001), while having a direct negative effect on change in sitting time (*p* < 0.0001). Baseline WC had a direct negative effect on change in WC (*p* < 0.0001). Finally, change in MVPA had a direct negative effect on change in WC.

**Table 3:** Structural equation models for the relationship between socioeconomic status, MVPA, and sitting time on changes in BMI and WC in urban black South African women

Effect of:	Outcomes:	Direct effects	Indirect effects	Total effects	Proportion of total effect mediated
SES^b^	BMI^c^ via MVPA^b^	0.022 (0.003; 0.041)*	0.0006 (−0.0022; 0.0035)	0.0226 (0.0038; 0.0413)*	0.03
	MVPA^c^ via BMI^b^	−3.81 (−6.92; −0.70)*	−0.39 (−4.84; 4.06)	−4.20 (−9.63; 1.22)	0.1
	Sitting^c^ via WC^b^	1.08 (−0.83; 2.98)	0.07 (−4.73; 4.86)*	1.14 (−4.02; 6.30)	0.06
MVPA^b^	BMI^c^ via MVPA^c^	−0.0004 (−.001; 0.000)	0.0003 (0.0003; 0.0003)***	−0.0001 (− 0.0007; 0.0004)	0.43^a^
	WC^c^ via MVPA^c^	−0.002 (− 0.003; − 0.0003)*	0.01 (0.001; 0.001)***	−0.001 (− 0.002; 0.001)	10^a^
	MVPA^c^ via BMI^b^	−0.87 (− 0.92; − 0.82)***	−0.0003 (− 0.0009; 0.0004)	−0.87 (− 0.92; − 0.82)***	0.0003
	MVPA^c^ via WC^b^	− 0.87 (− 0.92; − 0.82)***	−0.0008 (− 0.004; 0.002)	−0.87 (− 0.92; − 0.82)***	0.001^a^
Sitting^b^	BMI^c^ via Sitting^c^	− 0.0002 (− 0.0009; 0.001)	0.0001 (0.0001; 0.0002)***	−0.00002 (− 0.0008; 0.001)	5.00^a^
	WC^c^ via Sitting^c^	−0.0004 (− 0.002; 0.001)	0.0006 (0.0005; 0.001)***	0.0002 (− 0.002; 0.002)	3.00
	Sitting^c^ via WC^b^	−0.84 (− 0.86; − 0.81)***	0.0004 (− 0.0005; 0.001)	−0.84 (− 0.86; 0.81)***	0.001^a^
WC^b^	WC^c^ via MVPA^c^	−0.12 (− 0.18; − 0.05)***	0.003 (− 0.002; 0.09)	−2.45 (− 6.56; 1.67)	0.001^a^
	WC^c^ via Sitting^c^	−0.11 (− 0.17; − 0.05)***	0.0006 (− 0.001; 0.002)	−0.11 (− 0.17; − 0.05)***	0.01^a^
MVPA^c^	WC^c^	− 0.001 (− 0.003; − 0.00004)*		−0.001 (− 0.003; − 0.00004)*	

Data in parentheses are 95% CIs; All models were adjusted for age

*MVPA* moderate to vigorous physical activity, *BMI* body mass index, *SES* socioeconomic status, *WC* waist circumference

*P < 0.05; ***P < 0.001

^a^Assessed using the absolute values for both indirect and direct effects

^b^Baseline values

^c^Change values

**Fig. 1:**
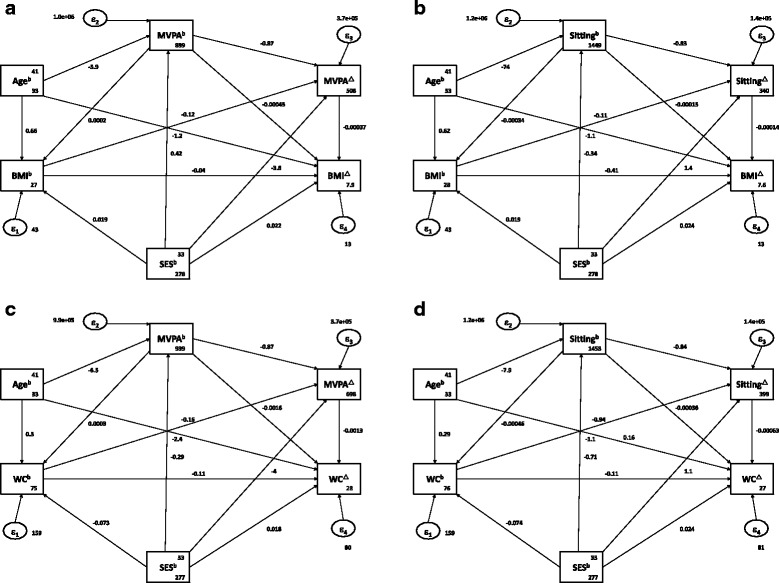
**a**, **b**, **c** and **d**: Path diagram of the association of socioeconomic status (SES) with change in body mass index (BMI) via baseline (**b**) and change (^∆^) in moderate-vigorous physical activity (MVPA) (**a**) and via sitting time^b∆^ (**b**); and the association of baseline SES with change in waist circumference (WC) via MVPA ^b∆^ (**c**) and sitting time ^b∆^ (**d**)

The values indicated that the model was acceptable and best fit for the model (Table 4, Fig. 1). The SEM model for SES and baseline MVPA on change in BMI resulted in a model with a CFI value of 1.00, RMSEA value of 0.000, and SRMR value of 0.01 (Table 4, Fig. 1a). Table 4 and Fig. 1b represent the SEM model for the association of baselines SES and sitting on change in BMI. The CFI (1.00), RMSEA (0.000), and SRMR (0.01) indicated a good fit for the SEM model. The SEM for SES and baseline MVPA on change in waist is shown in Table 4 and Fig. 1c, and is considered to be the best fit model (CFI = 1.00; RMSEA = 0.000; SRMR = 0.01).The SEM model for baseline SES and baseline sitting on change in WC likewise presented a good fit with CFI = 1.00; RMSEA = 0.000; and SRMR = 0.01 (Table 4 and Fig. 1d).

**Table 4:** Fit indices for the structural equation model

Model	*X* ^2^	Prob > *X*^2^	RMSEA	CFI	TLI	SRMR	CD
SES and MVPA on BMI^a^	1.77	0.88	0.00	1.00	1.01	0.006	0.06
SES and sitting time on BMI^a^	3.57	0.61	0.00	1.00	1.00	0.006	0.05
SES and MVPA on WC^a^	1.02	0.91	0.00	1.00	1.02	0.005	0.04
SES and sitting time on WC^a^	3.16	0.53	0.00	1.00	1.00	0.006	0.03

*RMSEA* Root Mean Square Error of Approximation, *CFI* Comparative Fit Index, *TLI* Tucker Lewis Index, *SRMR* Standardized Root Mean Squared Residual, *CD* Coefficient of Determination, *BMI* body mass index, *WC* waist circumference, *SES* socioeconomic status

^a^Change values; *X*^2^ = Chi-squared test of fit model

## Discussion

The purpose of this longitudinal study was to determine the extent to which physical activity or sitting time mediate the association between SES and changes in BMI and central adiposity. The obesity trends observed in this study population are consistent with changes in adiposity in African women living in other sub-Saharan African countries [4]. This study confirms previous reports of increases in adiposity over time in African women, and shows how household asset derived SES influences these changes. We have shown that SES is associated with lower MVPA, higher sitting time, and consequently higher changes in BMI, suggesting that this cohort of African women is still experiencing some degree of economic transition. The results demonstrating the direct and indirect effects of SES and physical activity measures on changes in adiposity are the first from an African study, and could only be uncovered using structural equation modelling. Public health initiatives should be made aware of these study findings, particularly as obesity continues to increase with economic improvement in the sub-Saharan African region.

Research from South Africa [13–15] and other sub-Saharan African countries [16, 17] have shown that SES is positively associated with BMI. A country-wide demographic study in South Africa also showed that in all ethnic groups, men and women with some form of schooling have higher BMI values than those who have not attended school [29]. The findings of the present study confirm the current evidence, suggesting that adiposity increases with economic growth. In contrast, existing work from high-income countries shows that SES is inversely related with health outcomes [30, 31], while in non-African developing countries the relationship between SES and BMI does not appear to be constant [32]. In a recent study of young adult black South African women, SES was observed to have a direct effect on BMI in the urban population but not in the rural population [33]. These data support the phenomenon of within-country variation in the impact of SES on body adiposity, and can further vary according to region-specific sociodemographic context and geographic location [34]. Given the well-known nutritional and epidemiological transitions currently occurring in South Africa [35], our finding showing that SES was associated with lower change in MVPA is not surprising and is consistent with evidence from cross sectional [19, 33] and longitudinal studies [36, 37]. In rural South African populations, SES is positively associated with physical activity in adolescents [38] but not in adult women [33]. The urban-rural variation in socioeconomic status could explain the reason for the lack of association between SES and physical activity in rural dwelling women [33].

Consistent with other studies of African women, our data shows that urban dwelling African women have a high level of physical activity [38–40]. These studies show that rural dwelling African women have higher amounts of MVPA compared with urban dwellers. The transition to urban hubs results in acquiring comparatively less energy-demanding jobs than in the rural settings [41], and this may be another reason for the greater difference in volume of physical activity observed in urban African women [33]. Women in urban settings sit for longer periods as a consequence of less physically demanding work, and this could explain the indirect positive effect between baseline sitting and change in BMI and central fat demonstrated in the present study. Interestingly, these effects were mediated by greater increases in sitting time. Previous studies of the cohort in the present study have shown that high sitting is associated with obesity and associated cardiometabolic diseases [42]. The literature indicates an increasing pattern of obesity with continuing urbanisation in African populations [4]. Public health interventions can address the problem by targeting sedentary behaviour, particularly during travel and occupation time. The recent work by Ekelund et al. for example [18], demonstrates that reduced sitting time is associated with lower risk for all-cause mortality by improving cardiovascular health.

Baseline MVPA was shown to have a direct negative effect on change in central fat, which is consistent with other studies [43], and suggests that physical activity has a protective effect against excess fat accumulation. Our findings also reveals an indirect positive effect between baseline MVPA and measures of change in adiposity, driven by the direct negative effect between baseline MVPA and change in MVPA. This suggests that change in MVPA may a potential confounder as this study population ages, possibly explained by the continuous rural-urban shift as noted in a recent of younger adult African women [33]. We agree with this explanation, however the literature also suggests that eating behaviour and other potential correlates of obesity can change during the transition period [32], emphasising the need for additional investigation of behaviour in the current study population. Further, evidence demonstrates that in spite of transitioning populations obtaining sufficient levels of weekly physical activity, obesity levels continue to increase [44]. In the current study, the prevalence of obesity and central fat increased significantly in the study population from baseline to 10-year follow-up, by 15.2% and 18% respectively, however, the weekly MVPA was high, 50 mins./day at baseline and 34.3 mins./day at follow-up. In the context of a growing population of ageing African women, it is therefore paramount for policy makers to consider incorporating other forms of behavioural modification such as lowering caloric consumption to decrease weight gain.

The SEM approach used in this study has a number of strengths, particularly in understanding the increasing risk of obesity in African women. SEM has been used widely in cross-sectional and longitudinal studies, and despite being seldom used in epidemiological research this approach has applications in path analysis [45]. Sample size is the main limitation related to the SEM approach, and as evidence recommends a minimum of 200 observations, this was not a concern [46]. In this longitudinal study, SEM enabled the measurement of direct, indirect, and total effects, in addition to interpreting the underlying pathways of increasing body composition by detecting or removing the possible mediators involved in the accumulation of fat in women over a 10-year follow-up period. In this study we have therefore observed novel and scientifically plausible pathways which previous traditional multivariable regression analyses of this study population have not uncovered [20]. In addition, this study has highlighted the direct and indirect effects in the relationship between SES and physical activity determinants of change in adiposity in African women [20, 42].

The main limitation of this study was that measurements were made only at baseline and 10 years later due to limited resources, however the 10-year follow-up period did allow us to observe the long term influence of baseline determinants and changes in appropriate variables on body anthropometry in an African population with high obesity risk. This study did not control for eating behaviour as data were not available, however this data are currently being collected with the aim of understanding the role of caloric intake in obesity in African women. In addition, future studies should collect data on other potential behavioural predictors of adiposity. The physical activity measures obtained in the present study are based on the self-reported and validated global physical activity questionnaire [25]. Despite the methodological differences, our findings are similar to studies using objectively measured physical activity [43]. Better methods such as MRI can provide more definitive data on adiposity compared with BMI, however, it is still acknowledged to be one of the simplest proxy indicators of fat and is widely used in epidemiological studies [4, 5].

## Conclusions

In conclusion, this study demonstrates that the physical activity profile of urban African women is reasonable; however the threat of obesity is high and likely to continue increasing with advancing age. Changes in BMI and central fat can be minimised by maintaining or increasing energy expenditure through physical activity, in addition to limiting the negative impact of sedentary behaviour. The influence of other behavioural determinants should however also be considered in any future initiatives to address obesity in this vulnerable community of ageing African women.

## Additional file

Additional file 1: Sensitivity analysis on baseline study characteristics for participants in and out of the study. (DOCX 16 kb)
