# Supplementary material for: Influence of socioeconomic status on changes in body size and physical activity in ageing black South African women
Source: Eur Rev Aging Phys Act. 2018 Apr 26;15:6. doi: 10.1186/s11556-018-0196-8 (PMC5921976; doi:10.1186/s11556-018-0196-8)
Supplement: Supplementary file 1 — Sensitivity analysis on baseline study characteristics for participants in and out of the study. (DOCX 16 kb) [file 11556_2018_196_MOESM1_ESM.docx]

Additional file 1. Sensitivity analysis on baseline study characteristics for participants in and out of the study

| Baseline study characteristics | In the study  (n=518) | Out of the study  (n= 731) | P-value |
| --- | --- | --- | --- |
| Height (cm) | 158.3 ± 0.2 | 158.1 ± 0.2 | 0.72 |
| Weight (kg) | 76.1 ± 0.7 | 76.4 ± 0.68 | 0.36 |
| WC (cm) | 86.9 ± 0.5 | 87.8 ± 0.5 | 0.11 |
| Hip (cm) | 113.2 ± 0.6 | 112.3 ± 0.5 | 0.88 |
| BMI (kg/m^2^) | 30.4 ± 0.3 | 30.6 ± 0.3 | 0.27 |
| MVPA | 657.3 ± 44.0 | 650.9 ± 34.8 | 0.55 |
| Sitting time | 1116.6 ± 47.6 | 1213.8 ± 39.95 | 0.06 |
| SES | 33.1 ± 0.7 | 32.2 ± 0.7 | 0.82 |

Data presented as mean ± SD; *BMI* body mass index; *MVPA* moderate-vigorous physical activity; *SES* socioeconomic status; *WC* waist circumference
